# Detection and Serological Evidence of European Bat Lyssavirus 1 in Belgian Bats between 2016 and 2018

**DOI:** 10.3390/tropicalmed9070151

**Published:** 2024-07-05

**Authors:** Inne Nauwelaers, Claudia Van den Eynde, Sanne Terryn, Bob Vandendriessche, Wout Willems, Daan Dekeukeleire, Steven Van Gucht

**Affiliations:** 1 Viral Diseases Unit, Sciensano, 1000 Brussels, Belgium; inne.nauwelaers@sciensano.be (I.N.); sanne.terryn@sciensano.be (S.T.); 2 Natuurpunt Studie, Vleermuizenwerkgroep, 2800 Mechelen, Belgiumwout.willems@natuurpunt.be (W.W.);; 3 Research Institute for Nature and Forest, 1000 Brussels, Belgium

**Keywords:** European bat lyssavirus, rabies, surveillance, serology, PCR

## Abstract

Lyssaviruses are neurotropic viruses capable of inducing fatal encephalitis. While rabies virus has been successfully eradicated in Belgium, the prevalence of other lyssaviruses remains uncertain. In this study, we conducted a survey on live animals and passive surveillance to investigate the presence of lyssaviruses in Belgium. In 2018, a total of 113 saliva samples and 87 blood samples were collected from bats. Saliva was subjected to RT-qPCR to identify lyssavirus infections. Additionally, an adapted lyssavirus neutralisation assay was set up for the detection of antibodies neutralising EBLV-1 in blood samples. Furthermore, we examined 124 brain tissue samples obtained from deceased bats during passive surveillance between 2016 and 2018. All saliva samples tested negative for lyssaviruses. Analysis of the blood samples uncovered the presence of lyssavirus-neutralising antibodies in five bat species and 32% of samples with a wide range depending on bat species, suggesting past exposure to a lyssavirus. Notably, EBLV-1 was detected in brain tissue samples from two *Eptesicus serotinus* specimens collected in 2016 near Bertrix and 2017 near Étalle, confirming for the first time the presence of EBLV-1 in Belgium and raising awareness of the potential risks associated with this species of bats as reservoirs of the virus.

## 1. Introduction

After rodents, bats are the most species-rich group of mammals, and many species remain threatened [1]. They play a key role in the delivery of ecosystem services such as the control of insect species that are agricultural pests [2]. However, bats are also significant to public health as potential hosts of viruses, such as lyssaviruses.

*Lyssavirus rabies* (RABV) is one of 18 recognised species in the Lyssavirus genus according to the International Committee on Taxonomy of Viruses [3]. It has been eliminated in Belgium since 2001 after sustained domestic animal and wildlife vaccination campaigns, although the rabies-free status was briefly withdrawn after the illegal import of an infected dog in 2007 [4]. 

Several species of lyssavirus can cause fatal encephalitis in mammals. In Europe, *Lyssavirus hamburg (*EBLV-1), *Lyssavirus helsinki (*EBLV-2), *Lyssavirus bokeloh* (BBLV) [5], *Lyssavirus lleida* (LLEBV) [6], *Lyssavirus caucasicus* (WCBV) [7], *Lyssavirus kotalahti* (KBLV) [8] and the virus *Divača bat lyssavirus* (DBLV)*,* for which no species name has been decided yet [9], have been detected in bats. EBLV-1 is the most widespread bat lyssavirus in Europe and the virus and/or its antibodies have been detected in neighbouring countries like France (up to 50% of bats had antibodies in a single infected colony of *Eptesicus serotinus*) [10,11], Germany [12,13], the Netherlands [14,15] and the United Kingdom [16]. In Spain, up to 20% of tested bats were positive for antibodies against EBLV-1 [17], and in Eastern Europe, antibodies against lyssaviruses were demonstrated in seven different bat species on samples collected between 2014 and 2018 [18]. The rabies-free status of countries always refers to freedom of rabies in terrestrial animals, and therefore the circulation of lyssaviruses in bats does not influence the rabies-free status of a country. 

Although most human infections with lyssaviruses are caused by dog bites resulting in an infection with (classical) rabies virus [19], bats are believed to be the evolutionary hosts of lyssaviruses [17,20,21,22]. It has been shown by Robardet et al. that they can sustain infection with EBLV-1 with variable mortality, in contrast to other animals in which infection with lyssaviruses is inevitably fatal [10,23]. Lyssaviruses can be transmitted to other mammals, including humans, through licking open wounds, biting or scratching. This happens frequently in the Americas with RABV. In Europe, RABV is not circulating in bats, but there have been a few documented cases involving other lyssaviruses [24,25,26]. Most human cases from bats in Europe are caused by EBLV-1 or 2 [27].

In Europe, bat lyssaviruses seem to have specific bat species as hosts, namely *E. serotinus* and *isabellinus* and *Myotis dasycneme* for EBLV-1, *M. daubentonii* and *M. dasycneme* for EBLV-2, *M. nattereri* for BBLV, *Miniopterus schreibersii* for LLEBV [15,28] and WCBV [7], *M. brandtii* for KBLV, which has only been described in Finland [8,19], and *M. capaccinii* for DBLV, which has only been described in Slovenia [9].

The current prevalence of EBLV-1 in Belgium is insufficiently studied. Several species of bats live in Belgium, including *E. serotinus*, *M. daubentonii*, *M. dasycneme* and *M. nattereri,* which are known carriers of EBLV-1, EBLV-2 and BBLV, respectively. This study aimed to determine whether lyssaviruses are prevalent in Belgium via both passive surveillance and, for the first time, a survey in live animals. The results allow us to estimate the risk of zoonotic lyssaviruses in Belgium more accurately and can impact further strengthening of surveillance efforts for public health purposes.

## 2. Materials and Methods

### 2.1. Ethics Statement

All experimental procedures were approved by the Ethical Committee of Sciensano (advice number 2018041-01). Bat capture and handling was carried out under guidelines and license from Natuurpunt, Agentschap Natuur en Bos and the Flemish government (reference ANB/BL/FF-V18-00095).

### 2.2. Sampling of Bats

**Figure 1:**
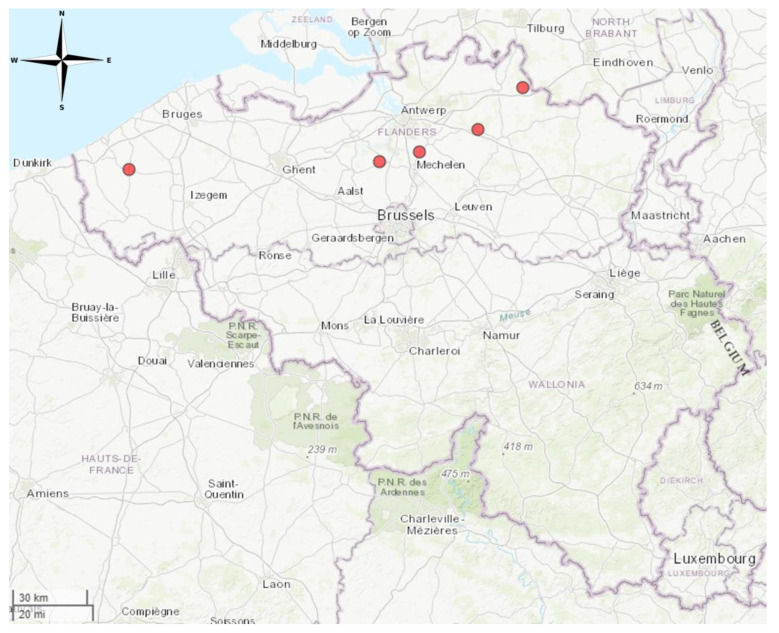
Collection sites in Flanders where bats were captured for saliva and blood collection and sampling. Sampling sites indicated on the map from left to right are Diksmuide, Liezele, Duffel, Herentals and Arendonk. Map created in R using the mapview package (https://cran.r-project.org/web/packages/mapview/index.html, accessed on 7 June 2024).

For the survey in live animals, five sampling sites that were never sampled before for lyssavirus surveillance were selected in Flanders. The sites were attended 6 h per night and were located at Arendonk (three nights), Diksmuide (one night), Duffel (four nights), Herentals (one night) and Liezele (one night) (Figure 1). Apart from a foraging location (Herentals), all other sites were near known hibernation sites for bats. Bats were captured at these sites for sample collection during the swarming season between mid-July and mid-October of 2018. During this period, different species of bats from different summer colonies visited hibernation sites to “swarm”, a behaviour linked to prospecting suitable hibernation sites and to mating [29,30].

At all locations, bats were captured using bat mist nets (Ecotone, Poland) that were kept under constant surveillance. Bats were disentangled immediately after capture and then individually placed in lightweight cotton holding bags until inspection and sampling. Upon inspection, bat species were determined based on morphological characteristics by an expert in the field [1]. Captured bats were marked with a non-toxic marker to avoid repeated sampling if they were recaptured. Bats were released immediately after sampling. Recaptured bats were released immediately as well.

Both blood and saliva samples were collected for further research. Saliva samples were collected from the tongue using cotton swabs and stored at 4 °C in 200 µL of sterile phosphate-buffered saline (PBS). Blood samples from the interfemoral vein medial of the femur [31] were collected on filter paper as no more than one drop of blood could be obtained to ensure the safety of the bats. Saliva samples were used for real-time quantitative (RT-q) PCR to detect the presence of lyssaviruses. Blood samples were used to detect the presence of antibodies neutralising EBLV-1 using the method described below.

Passive surveillance consisted of bats being brought in from wildlife rehabilitation centres that received animals that were severely weakened and died or were found dead. Bats from all parts of Belgium were included if the date and place of discovery was available and the cadaver was in a good enough state for sample collection. Brain tissue was collected from the bats for RT-qPCR testing to determine the presence of different lyssaviruses, as specified below.

### 2.3. Detection of Lyssaviruses

Detection of lyssavirus RNA was conducted using a pan-lyssa RT-qPCR according to the method described by Fischer et al. [21]. Some samples were tested with the fluorescent antigen test (FAT) instead according to protocol of the WHO [32].

Prior to the RT-qPCR, total RNA was extracted using the Qiagen RNeasy kit (Qiagen, Hilden, Germany) according to the manufacturer’s instructions, using 50 µL of PBS in which cotton swabs were placed mixed with 300 µL of lysis buffer (Qiagen, Hilden, Germany).

The RT-qPCR was performed with primers JW12 and N145-165 [21], and extraction control was performed using primers VETINHR1 and VETINHF2 targeting 18S. The enzyme used was Q-script One Step RT (Quantabio, Beverly, MA, USA). This RT-qPCR can detect RABV, Lagos bat virus, BBLV, Mokola virus, Duvenhage virus, Australian bat lyssavirus, EBLV-1 and EBLV-2 [21]. Positive samples were sent to the European Reference Centre for Rabies (ANSES, Nancy, France) to be sequenced for genotyping.

### 2.4. Detection of Past EBLV-1 Infection through Modified Seroneutralisation Assay

Filter paper blood samples were incubated overnight in 200 µL of sterile PBS. The solution was tested for the presence of antibodies against EBLV-1 using the modified rapid fluorescent focus inhibition test (RFFIT) based on the Manual of Diagnostic Tests and Vaccines for Terrestrial Animals [33]. To increase specificity for EBLV-1-neutralising antibodies, an in-house modified neutralisation assay was designed using EBLV-1 virus instead of the standard CVS-11 RABV. The EBLV-1 strain used was called AF-2010, cultured from a positive bat from Spain [26]. Briefly, 1 in 3 serial dilutions of the sample were incubated at 37 °C for 90 min with 217.2 Median Cell Culture Infectious Dose (CCID_50_)/mL of EBLV-1. To control consistency between tests, the same viral dose of EBLV-1 had to neutralise the positive control to the same dilution for each test. For the positive control, OIE International Standard Serum for Rabies from dog origin (ANSES, LOT 2014-1) was diluted to 0.5 IU/mL for RABV neutralisation and used as EBLV-1 control as well. Antibodies bound the virus if any were present. Then, baby hamster kidney (BHK-21) cells (ATCC CCL-10, ordered from DSMZ, Leibniz, Germany, in 2013) were added to the mixture and incubated for 24 h. Infected BHK-21 cells were stained with fluorescent anti-nucleocapsid antibody and microscopically counted. The dilution that inhibited 50% of infection was determined. The cut-off value for positivity was chosen at a reciprocal titre of ≥27 to ensure no false positive reactions were read, as previously applied by Harris et al. [34]. The only goal was to detect the presence of antibodies that neutralise EBLV-1, not to determine exact titres. Cross-reaction of antibodies against other lyssaviruses that also neutralise EBLV-1 cannot be excluded.

## 3. Results

### 3.1. Bat Prevalence in Flanders

#### 3.1.1. Life Animal Survey

During the survey in live animals in 2018, bats were captured at five sampling sites in Flanders, Belgium, none of which were sampled before (Figure 1). Five different species were identified. A total of 120 bats were captured. Of these, 79 were *M. daubentonii*, 12 were *M. emarginatus*, 3 were *M. mystacinus*, 3 were *M. nattereri* and 23 were *Plecotus auritus*. A full list of samples can be found in the Supplementary Materials (Table S1).

In total, 120 bats were captured of which 113 saliva samples and 87 blood samples were collected (Table 1). Multiple species were captured at 4 of the 5 sites, as indicated in Table 1.

#### 3.1.2. Passive Surveillance

**Figure 2:**
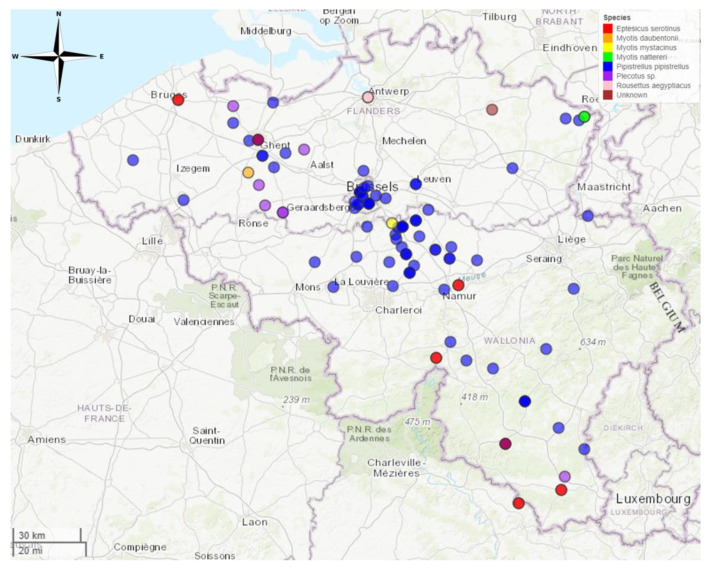
Locations in Belgium where bats were found during passive surveillance. Map created in R using the mapview package (https://cran.r-project.org/web/packages/mapview/index.html, accessed on 7 June 2024).

Passive surveillance was performed on bats brought in from wildlife rehabilitation centres between 2016 and 2018. In total, 133 bats were collected. A full list of samples can be found in the Supplementary Materials (Table S2). Nine samples did not have location information and/or could not be analysed due to the degraded state of the samples. A total of 124 animals were included in this study. Of these, 32 (25.8%) were from the Flemish region, 60 (48.4%) were from the Walloon region and 31 (25.0%) were from the Brussels region. One bat had an unknown origin (0.8%) (Figure 2).

Out of 124 animals, 98 (79.0%) were *Pipistrellus pipistrellus*, 13 (10.5%) were *E. serotinus*, 2 (1.6%) were *M. nattereri*, 1 (0.8%) was *M. daubentonii*, 1 (0.8%) was *M. mystacinus* and 9 (7.3%) could not be identified. No saliva or blood samples could be obtained from these bats due to the condition in which they were collected, so only brain samples were tested.

*Eptesicus serotinus* was found at 7 locations. *Myotis daubentonii* was found at one location (Gavere). *Myotis mystacinus* was found at one location (Lasne). *Myotis nattereri* was found at one location (Kinrooi). *Pipistrellus pipistrellus* was found at 58 different locations. *Plecotus sp.* were found at 6 different locations. *Rousettus aegyptiacus* was found at one location (illegally imported through Antwerp airport) (Table 2).

In 2016, 48 bats were brought in of which 7 could not be analysed, so 41 were tested. In 2017, 43 bats were brought in and all were tested. In 2018, 43 bats were brought in of which 2 could not be tested, so 41 were included in this study.

### 3.2. Detection of Lyssavirus in Belgian Bats

Saliva samples taken during the survey in live animals were tested for the presence of lyssaviruses using RT-qPCR. In total, 113 samples were tested of which zero tested positive for any lyssavirus. The numbers of samples tested per location were 12, 11, 77, 7 and 6 for Arendonk, Diksmuide, Duffel, Herentals and Liezele, respectively.

**Figure 3:**
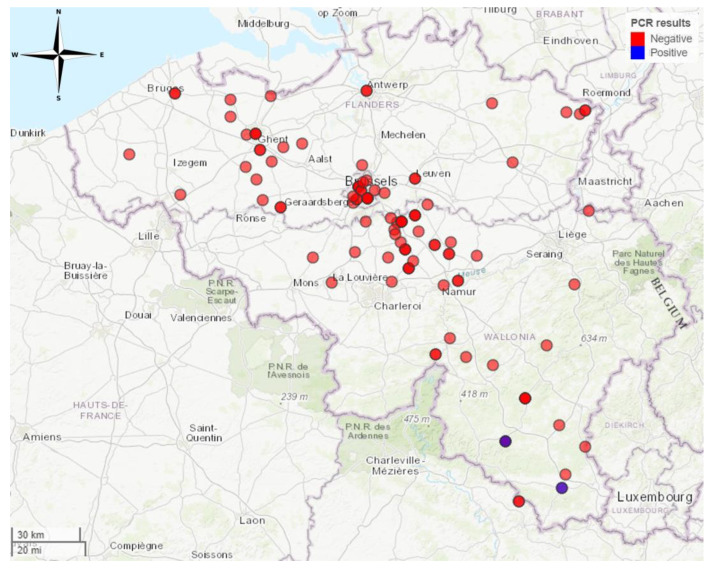
Locations in Belgium where bats were found during passive surveillance. Two *E. serotinus* bats tested positive for EBLV-1, one in 2016 in Bertrix and one in 2017 in Étalle, which is 30 km southeast of Bertrix. Map created in R using the mapview package (https://cran.r-project.org/web/packages/mapview/index.html, accessed on 7 June 2024).

Samples of passive surveillance were tested using the same RT-qPCR and/or by FAT. In 2016, one bat tested positive for a lyssavirus by RT-qPCR methods out of 41 tested cadavers (2%). The severely weakened and immobile *E. serotinus* was brought to the Belgian National Reference Laboratory on 28 September 2016 after a biting incident with a hiker in Bertrix (Figure 3). The hiker received post-exposure prophylaxis afterwards and remained in good health.

In 2017, one other *E. serotinus* bat tested positive for lyssavirus by RT-qPCR out of 43 cadavers (2%). It was found on 16 October 2017 in Étalle (Figure 3), which is about 30 km southeast of Bertrix. Upon discovery of the weakened bat, a bat care centre in Bertrix was contacted and no human exposure was reported. Sequencing of the N gene by the European Reference Centre for Rabies (ANSES) confirmed EBLV-1 infection in both cases. Not enough sample was left to sequence the full genome.

In 2018, none of the 41 samples (0%) tested positive by RT-qPCR.

### 3.3. Modified Neutralisation Assay Shows Presence of EBLV-1-Neutralising Antibodies

Antibody assays can detect immune responses mounted against previous infections. Bats captured during the survey in 2018 were tested using this method. Since blood cannot be drawn from bat cadavers, it was not possible to test passive surveillance samples for antibody presence.

Out of 87 bats from which blood samples were collected, 32% tested positive for EBLV-1-neutralising antibodies with a range of 0% to 67% depending on the bat species (Table 2). Antibodies neutralising EBLV-1 might be mounted against EBLV-1 or against another lyssavirus and cross-protect against EBLV-1. The causative agent is therefore a lyssavirus but cannot be proven to be EBLV-1 specifically. A total of 16 specimens of *M. daubentonii* and 8 specimens of *P. auritus* tested positive for antibodies neutralising EBLV-1. Notably, there were also two specimens of *M. emarginatus* and two specimens of *M. mystacinus* that tested positive for antibodies neutralising EBLV-1.

In Arendonk, 27% (3/11) of bats showed antibodies neutralising EBLV-1. In Diksmuide, 18% (2/9) had antibodies. In Duffel, 33% (18/55) showed antibodies neutralising EBLV-1. In Herentals, two out of four were positive. In Liezele, three out of six showed presence of antibodies.

Overall, EBLV-1-neutralising antibodies could be detected in all bat species apart from *M. nattereri*. The number of positive samples ranged from 22% for *M. emarginatus* to 67% for *M. mystacinus*. The numbers were not large enough to infer conclusions about the presence of lyssaviruses per bat species (Table 2).

## 4. Discussion

The primary objective of this study centred upon the detection of *European Bat Lyssavirus 1* (EBLV-1) as well as the detection of antibodies directed against this virus among bat populations in Belgium. EBLV-1 was successfully identified for the first time in Belgium during the years 2016 and 2017 in two *E. serotinus* specimens, and antibodies were detected at varying levels in different bat species. This goal was accomplished through the combined implementation of a survey in live animals and passive surveillance methodologies. It needs to be noted that antibodies neutralising EBLV-1 might be mounted against other lyssavirus and are detected due to cross-neutralisation.

Passive surveillance between 2016 and 2018 exclusively allowed for the testing of deceased bats. RT-qPCR and/or FAT testing was conducted on cerebral tissue extracted from bat cadavers. One sample did exhibit positive results for EBLV-1 through both RT-qPCR and FAT testing in the year 2016. This specific sample originated from an *E. serotinus* collected in Bertrix, situated within the southern part of Belgium. This particular bat specimen showed no wounds, and despite manifesting signs of despondency, it exhibited an aggressive response when subjected to handling. Subsequent to this initial case, a second *E. serotinus* yielded a positive outcome in RT-qPCR analysis one year later and was found in Étalle, situated approximately 30 km southeast of Bertrix. Although the possibility exists that these bats belonged to the same colony, this could not be verified. Serological and RNA evidence for EBLV-1 in this species has already been described by Robardet et al. for the northeast of France, which is not far from the Belgian border [10].

In the region of Flanders, five different bat species, namely *M. daubentonii*, *M. emarginatus*, *M. mystacinus*, *M. nattereri* and *P. auritus*, were captured during a survey in 2018. The investigations encompassed collection of fluid samples, including saliva and blood, from apparently healthy adult bats. Salivary specimens obtained from animals captured in live conditions yielded negative results across all samples for all tested lyssaviruses. This observation might be attributed to the intermittent nature of viral shedding, which ceases following seroconversion, as highlighted by Fooks et al. [35]. As such, the efficacy of RT-qPCR in detecting viral presence within saliva samples is inherently limited. These bats also showed a marked mycological diversity, including a new *Pseudogymnoascus* species, as described by Becker et al. [36].

Blood samples collected in 2018 were utilised for the purpose of detecting neutralizing antibodies specific to EBLV-1. EBLV-1-neutralising antibodies were detected in 32% of bats, ranging from 0% to 67% depending on bat species. *Plecotus auritus* samples have been shown before to be positive in neighbouring countries [10,13,37], and this study has confirmed the presence of antibodies against EBLV-1 in this species in Belgium.

This high percentage of antibodies in blood samples implies that infection with lyssaviruses is not uncommon in several of the captured bat species.

Remarkably, a notable proportion of blood samples from *M. daubentonii* tested positive as well, which is particularly significant due to the previous recognition of *M. daubentonii* as a host for EBLV-2 in other countries [28]. It is therefore most likely that these animals have antibodies against EBLV-2 yet are cross-neutralising against EBLV-1.

*Myotis emarginatus* and *M. mystacinus* also tested positive for EBLV-1-neutralising antibodies, which has not yet been shown before. It should be noted that the sample quantities were insufficient to also allow testing for antibodies against other lyssaviruses, but it is most likely that the antibodies mounted by these species are targeting other lyssaviruses and are cross-neutralising EBLV-1 rather than antibodies mounted against EBLV-1 specifically. Consequently, cross-reactivity of antibodies against other lyssaviruses from the same phylogroup remains likely, which is in line with the insights put forth by Weir et al. [37] and Inoue et al. [38]. Future research will focus on alternate blood sampling methods to allow for more tests and to be able to distinguish between antibodies targeting specific lyssaviruses. This will support further studies to yield more insight into the different lyssaviruses in Belgium.

In conclusion, this is the first study that shows that bats in Flanders have antibodies neutralising EBLV-1, suggesting the virus is circulating in Belgium, like it is in neighbouring countries. However, cross-neutralising antibodies against other lyssaviruses from the same phylogroup cannot be excluded. Furthermore, two cases of active EBLV-1 infection were detected in *E. serotinus* specimens in 2016 and 2017, confirming its presence in at least one bat species. Surveillance efforts should be strengthened to further monitor the current situation of lyssaviruses in Belgium, to be able to distinguish between them and to follow up on its effects on important and threatened bat populations. Belgium is already declared free of rabies in terrestrial mammals and therefore meets the objective of a rabies-free Europe. However, this research shows that rabies in free-flying mammals should be taken into account, in addition to it currently being a blind spot in the mapping of lyssaviruses in Europe.

## Figures and Tables

**Table 1:** Overview of samples taken during the survey in live animals. Samples were taken from five locations in Flanders, Belgium. Saliva and blood samples were taken where possible.

Location/Bat Species	Number of Bats Captured	Number of Saliva Samples Taken	Number of Blood Samples Taken
Arendonk	12	12	11
*M. emarginatus*	1	1	1
*M. nattereri*	1	1	1
*P. auritus*	10	10	9
Diksmuide	12	11	12
*M. mystacinus*	1	1	1
*M. daubentonii*	11	10	11
Duffel	83	77	54
*M. daubentonii*	64	63	40
*M. emarginatus*	3	3	3
*M. mystacinus*	1	1	1
*M. nattereri*	2	2	2
*P. auritus*	13	8	8
Herentals	7	7	7
*M. emarginatus*	7	7	7
Liezele	6	6	6
*M. daubentonii*	4	4	4
*M. emarginatus*	1	1	1
*M. mystacinus*	1	1	1

**Table 2:** Results of seroneutralisation assay for EBLV-1-neutralising antibodies per bat species. The causative agent can be any lyssavirus from phylogroup I as cross-protection cannot be excluded.

Bat Species	Number of Bats Tested	Number of Samples Positive for Antibodies (%)
*M. daubentonii*	55	16 (29%)
Diksmuide	11	1
Duffel	44	13
Liezele	4	2
*M. emarginatus*	9	2 (22%)
Arendonk	1	0
Duffel	3	0
Herentals	4	2
Liezele	1	0
*M. mystacinus*	3	2 (67%)
Diksmuide	1	1
Duffel	1	0
Liezele	1	1
*M. nattereri*	3	0 (0%)
Arendonk	1	0
Duffel	2	0
*P. auritus*	17	8 (47%)
Arendonk	9	3
Duffel	8	5
TOTAL	87	28 (32%)

## Data Availability

Data can be requested by contacting the corresponding author.

## Supplementary Materials

The following supporting information can be downloaded at https://www.mdpi.com/article/10.3390/tropicalmed9070151/s1, Table S1: Detailed list of information on the bats captured during surveillance at 5 sites in Belgium.; Table S2: Detailed list of information on the bats tested during passive surveillance which were collected from 124 sites in Belgium.
