# Supplementary material for: Detection and Serological Evidence of European Bat Lyssavirus 1 in Belgian Bats between 2016 and 2018
Source: Trop Med Infect Dis. 2024 Jul 5;9(7):151. doi: 10.3390/tropicalmed9070151 (PMC11281572; doi:10.3390/tropicalmed9070151)
Supplement: Supplementary file 1 [file tropicalmed-09-00151-s001.zip › Table S1 - Survey of live animals.pdf]

Table S1: Detailed list of information on the bats captured during active surveillance at 5 sites in Belgium.

Bat species	Location	Saliva	PCR result	Blood	Titre	Dilution	Antibody result
M. emarginatus	Arendonk	Yes	Negative	Yes	0.31	9.73	Negative
Plecotus auritus	Arendonk	Yes	Negative	Yes	0.06	1.73	Negative
Plecotus auritus	Arendonk	Yes	Negative	Yes	0.06	1.73	Negative
Plecotus auritus	Arendonk	Yes	Negative	Yes	0.06	1.73	Negative
Plecotus auritus	Arendonk	Yes	Negative	Yes	0.06	1.73	Negative
Plecotus auritus	Arendonk	Yes	Negative	No			
Plecotus auritus	Arendonk	Yes	Negative	Yes	0.14	4.44	Negative
Plecotus auritus	Arendonk	Yes	Negative	Yes	0.94	29.20	Positive
Plecotus auritus	Arendonk	Yes	Negative	Yes	0.07	2.04	Negative
Plecotus auritus	Arendonk	Yes	Negative	Yes	1.27	39.66	Positive
Plecotus auritus	Arendonk	Yes	Negative	Yes	1.06	33.18	Positive
M. nattereri	Arendonk	Yes	Negative	Yes	0.07	2.04	Negative
M. daubentonii	Duffel	Yes	Negative	No			
M. daubentonii	Duffel	Yes	Negative	Yes	0.06	2.77	Negative
M. daubentonii	Duffel	Yes	Negative	Yes	0.75	37.54	Positive
M. daubentonii	Duffel	Yes	Negative	Yes	0.11	3.35	Negative
M. daubentonii	Duffel	Yes	Negative	Yes			Negative
M. daubentonii	Duffel	Yes	Negative	No			
M. daubentonii	Duffel	Yes	Negative	Yes	2.68	134.02	Positive
Plecotus auritus	Duffel	No		No			
M. daubentonii	Duffel	Yes	Negative	Yes	0.13	4.07	Negative
M. daubentonii	Duffel	Yes	Negative	Yes	1.17	58.26	Positive
M. daubentonii	Duffel	Yes	Negative	Yes	0.06	1.73	Negative
M. daubentonii	Duffel	Yes	Negative	No			
Plecotus auritus	Duffel	No		No			
M. daubentonii	Duffel	Yes	Negative	No			
M. mystacinus	Duffel	Yes	Negative	Yes	0.06	1.73	Negative
M. nattereri	Duffel	Yes	Negative	Yes	0.06	1.73	Negative
M. daubentonii	Duffel	Yes	Negative	No			
M. daubentonii	Duffel	Yes	Negative	No			
M. daubentonii	Duffel	Yes	Negative	No			
M. daubentonii	Duffel	Yes	Negative	No			
M. daubentonii	Duffel	Yes	Negative	No			
M. daubentonii	Duffel	Yes	Negative	No			
M. daubentonii	Duffel	Yes	Negative	No			
M. daubentonii	Duffel	Yes	Negative	No			
M. daubentonii	Duffel	Yes	Negative	No			
M. daubentonii	Duffel	Yes	Negative	Yes	0.17	5.20	Negative
M. daubentonii	Duffel	Yes	Negative	Yes	0.07	2.16	Negative
M. emarginatus	Duffel	Yes	Negative	Yes	0.06	1.73	Negative
M. daubentonii	Duffel	Yes	Negative	No			
M. daubentonii	Duffel	Yes	Negative	Yes	0.12	3.69	Negative
M. daubentonii	Duffel	Yes	Negative	Yes	0.27	13.59	Negative
M. daubentonii	Duffel	Yes	Negative	Yes	0.14	4.41	Negative
Plecotus auritus	Duffel	No		No			
M. daubentonii	Duffel	Yes	Negative	Yes	0.14	4.41	Negative
M. daubentonii	Duffel	Yes	Negative	Yes	0.71	35.53	Positive
M. daubentonii	Duffel	Yes	Negative	Yes	0.48	24.19	Negative

Plecotus auritus	Duffel	No		No			
M. daubentonii	Duffel	Yes	Negative	Yes	1.75	87.61	Positive
M. daubentonii	Duffel	Yes	Negative	No			
M. daubentonii	Duffel	Yes	Negative	No			
M. emarginatus	Duffel	Yes	Negative	Yes	0.18	5.49	Negative
M. daubentonii	Duffel	Yes	Negative	No			
M. daubentonii	Duffel	Yes	Negative	Yes	0.99	49.41	Positive
M. daubentonii	Duffel	Yes	Negative	No			
M. daubentonii	Duffel	Yes	Negative	Yes	0.15	7.32	Negative
M. daubentonii	Duffel	Yes	Negative	Yes	1.03	51.25	Positive
M. daubentonii	Duffel	Yes	Negative	Yes			Negative
M. daubentonii	Duffel	Yes	Negative	Yes	0.06	1.73	Negative
M. daubentonii	Duffel	Yes	Negative	Yes	0.06	1.81	Negative
Plecotus auritus	Duffel	Yes	Negative	Yes	1.17	58.26	Positive
Plecotus auritus	Duffel	Yes	Negative	Yes	1.17	58.26	Positive
M. daubentonii	Duffel	Yes	Negative	Yes	1.99	99.53	Positive
M. daubentonii	Duffel	Yes	Negative	Yes			Negative
M. daubentonii	Duffel	Yes	Negative	Yes	0.58	29.20	Positive
M. daubentonii	Duffel	Yes	Negative	No			
Plecotus auritus	Duffel	Yes	Negative	Yes	2.38	118.98	Positive
Plecotus auritus	Duffel	Yes	Negative	Yes	1.03	51.25	Positive
Plecotus auritus	Duffel	Yes	Negative	Yes	0.06	1.73	Negative
M. daubentonii	Duffel	Yes	Negative	Yes	2.54	126.96	Positive
M. daubentonii	Duffel	Yes	Negative	Yes	0.06	1.73	Negative
M. daubentonii	Duffel	Yes	Negative	Yes	0.06	1.73	Negative
M. daubentonii	Duffel	Yes	Negative	Yes	0.07	2.04	Negative
Plecotus auritus	Duffel	Yes	Negative	Yes	0.71	35.53	Positive
M. emarginatus	Duffel	Yes	Negative	Yes	0.06	1.91	Negative
M. daubentonii	Duffel	Yes	Negative	Yes	0.24	11.84	Negative
M. daubentonii	Duffel	Yes	Negative	Yes	0.13	3.95	Negative
M. daubentonii	Duffel	Yes	Negative	Yes	0.94	46.77	Positive
M. daubentonii	Duffel	Yes	Negative	Yes	0.15	4.60	Negative
M. daubentonii	Duffel	Yes	Negative	Yes	0.22	6.84	Negative
M. daubentonii	Duffel	Yes	Negative	Yes	0.23	7.32	Negative
M. daubentonii	Duffel	Yes	Negative	Yes	0.07	2.04	Negative
Plecotus auritus	Duffel	Yes	Negative	Yes	0.06	1.91	Negative
Plecotus auritus	Duffel	Yes	Negative	Yes	0.06	1.73	Negative
Plecotus auritus	Duffel	No		No			
M. daubentonii	Duffel	Yes	Negative	Yes	1.23	61.55	Positive
M. daubentonii	Duffel	Yes	Negative	Yes	0.33	16.47	Negative
M. daubentonii	Duffel	Yes	Negative	Yes	0.13	4.07	Negative
M. daubentonii	Duffel	Yes	Negative	Yes	0.06	1.73	Negative
M. daubentonii	Duffel	Yes	Negative	Yes	0.06	1.73	Negative
M. nattereri	Duffel	Yes	Negative	Yes	0.17	5.20	Negative
M. daubentonii	Duffel	Yes	Negative	Yes	2.20	109.91	Positive
M. daubentonii	Duffel	No		No			
M. daubentonii	Duffel	Yes	Negative	Yes	0.07	2.04	Negative
M. emarginatus	Herentals	Yes	Negative	Yes	0.39	12.21	Negative
M. emarginatus	Herentals	Yes	Negative	Yes	0.06	1.73	Negative
M. emarginatus	Herentals	Yes	Negative	Yes	3.19	99.53	Positive

M. emarginatus	Herentals	Yes	Negative	Yes	2.81	140.30	Positive
M. emarginatus	Herentals	Yes	Negative	No			
M. emarginatus	Herentals	Yes	Negative	No			
M. emarginatus	Herentals	Yes	Negative	No			
M. daubentonii	Diksmuide	Yes	Negative	Yes	4.50	140.30	Positive
M. daubentonii	Diksmuide	Yes	Negative	Yes	0.31	15.59	Negative
M. daubentonii	Diksmuide	Yes	Negative	Yes	0.06	1.73	Negative
M. daubentonii	Diksmuide	Yes	Negative	Yes	0.07	2.21	Negative
M. daubentonii	Diksmuide	No		Yes			Negative
M. daubentonii	Diksmuide	Yes	Negative	Yes	0.13	4.17	Negative
M. mystacinus	Diksmuide	Yes	Negative	Yes	4.30	134.02	Positive
M. daubentonii	Diksmuide	Yes	Negative	Yes	0.35	10.81	Negative
M. daubentonii	Diksmuide	Yes	Negative	Yes	0.16	4.85	Negative
M. daubentonii	Diksmuide	Yes	Negative	Yes	0.08	2.62	Negative
M. daubentonii	Diksmuide	Yes	Negative	Yes	0.38	11.84	Negative
M. daubentonii	Diksmuide	Yes	Negative	Yes	0.17	5.20	Negative
M. daubentonii	Liezele	Yes	Negative	Yes	3.53	109.91	Positive
M. mystacinus	Liezele	Yes	Negative	Yes	0.94	29.20	Positive
M. daubentonii	Liezele	Yes	Negative	Yes	0.54	16.70	Negative
M. daubentonii	Liezele	Yes	Negative	Yes	3.19	99.53	Positive
M. emarginatus	Liezele	Yes	Negative	Yes	0.50	15.59	Negative
M. daubentonii	Liezele	Yes	Negative	Yes	0.00	0.08	Negative
