# Supplementary material for: Detection and Serological Evidence of European Bat Lyssavirus 1 in Belgian Bats between 2016 and 2018
Source: Trop Med Infect Dis. 2024 Jul 5;9(7):151. doi: 10.3390/tropicalmed9070151 (PMC11281572; doi:10.3390/tropicalmed9070151)
Supplement: Supplementary file 1 [file tropicalmed-09-00151-s001.zip › Table S2 - Passive surveillance.pdf]

Table S2: Detailed list of information on the bats tested during passive surveillance which were collected from 124 sites in Belgium.

Species	Region	Postal Code	Parameter	Result
Pipistrellus Pipistrellus	Wallonië	5580	DirectIF	Negative
Pipistrellus Pipistrellus	Wallonië	5000	DirectIF	Negative
Pipistrellus Pipistrellus	Wallonië	1390	DirectIF	Negative
Pipistrellus Pipistrellus	Wallonië	1390	DirectIF	Negative
Pipistrellus Pipistrellus	Wallonië	6630	DirectIF	Negative
Pipistrellus Pipistrellus	Wallonië	7100	DirectIF	Negative
Pipistrellus Pipistrellus	Wallonië	5560	DirectIF	Negative
Pipistrellus Pipistrellus	Wallonië	6880	DirectIF	Negative
Pipistrellus Pipistrellus	Wallonië	6870	DirectIF	Negative
Eptesicus serotinus	Refused	Unknown		
Eptesicus serotinus	Refused	Unknown		
Pipistrellus Pipistrellus	Wallonië	1360	DirectIF	Negative
Pipistrellus Pipistrellus	Brussel	1000	DirectIF	Negative
Eptesicus serotinus	Refused	Unknown		
Pipistrellus Pipistrellus	Brussel	1170	DirectIF	Negative
Pipistrellus Pipistrellus	Brussel	1170	DirectIF	Negative
Pipistrellus Pipistrellus	Brussel	1170	DirectIF	Negative
Pipistrellus Pipistrellus	Wallonië	1342	DirectIF	Negative
Unknown	Vlaanderen	2490	DirectIF	
Pipistrellus Pipistrellus	Wallonië	1300	DirectIF	Negative
Pipistrellus Pipistrellus	Wallonië	1340	DirectIF	Negative
Pipistrellus Pipistrellus	Wallonië	7060	DirectIF	Negative
Unknown	Unknown	Unknown	DirectIF	
Unknown	Unknown	Unknown	DirectIF	
Unknown	Unknown	Unknown	DirectIF	
Unknown	Unknown	Unknown	DirectIF	
Pipistrellus Pipistrellus	Vlaanderen	3080	DirectIF	Negative
Pipistrellus Pipistrellus	Wallonië	1435	DirectIF	Negative
Pipistrellus Pipistrellus	Vlaanderen	9060	DirectIF	Negative
Pipistrellus Pipistrellus	Vlaanderen	9031	DirectIF	Negative
Plecotus sp.	Vlaanderen	9290	DirectIF	Negative
Pipistrellus Pipistrellus	Vlaanderen	9000	DirectIF	Negative
Pipistrellus Pipistrellus	Vlaanderen	9860	DirectIF	Negative
Pipistrellus Pipistrellus	Vlaanderen	9930	DirectIF	Negative
Pipistrellus Pipistrellus	Vlaanderen	9000	DirectIF	Negative
Pipistrellus Pipistrellus	Vlaanderen	9230	DirectIF	Negative
Pipistrellus Pipistrellus	Brussel	1180	DirectIF	Negative
Pipistrellus Pipistrellus	Wallonië	1325	DirectIF	Negative
Eptesicus serotinus	Wallonië	6880	PCR+DirectIF	Positive
Pipistrellus Pipistrellus	Vlaanderen	1701	DirectIF	Negative
Pipistrellus Pipistrellus	Brussel	1170	DirectIF	Negative
Pipistrellus Pipistrellus	Brussel	1140	DirectIF	Negative
Pipistrellus Pipistrellus	Brussel	1170	DirectIF	Negative
Pipistrellus Pipistrellus	Brussel	1000	DirectIF	Negative
Pipistrellus Pipistrellus	Brussel	1190	DirectIF	Negative
Pipistrellus Pipistrellus	Brussel	1150	DirectIF	Negative
Pipistrellus Pipistrellus	Brussel	1170	DirectIF	Negative
Pipistrellus pipistrellus	Brussel	1180	directIF	Negative
Pipistrellus pipistrellus	Wallonië	6880	directIF	Negative
Eptesicus serotinus	Wallonië	6823	directIF	Negative
Eptesicus serotinus	Wallonië	6823	PCR	Negative
Rousettus aegyptiacus?	Vlaanderen	2018	directIF	Negative
Pipistrellus pipistrellus	Wallonië	4210	directIF	Negative
Pipistrellus pipistrellus	Vlaanderen	8500	directIF	Negative
Pipistrellus pipistrellus	Wallonië	6870	directIF	Negative
Pipistrellus pipistrellus	Vlaanderen	8650	directIF	Negative
Plecotus sp.	Vlaanderen	9500	directIF	Negative
Plecotus sp.	Vlaanderen	9500	directIF	Negative
Pipistrellus pipistrellus	Brussel	1170	directIF	Negative
Pipistrellus pipistrellus	Brussel	1000	directIF	Negative

Pipistrellus pipistrellus	Wallonië	6870	directIF	Negative
Pipistrellus pipistrellus	Wallonië	6870	directIF	Negative
Pipistrellus pipistrellus	Wallonië	1450	directIF	Negative
Pipistrellus pipistrellus	Wallonië	1301	directIF	Negative
Pipistrellus pipistrellus	Wallonië	1450	directIF	Negative
Pipistrellus pipistrellus	Wallonië	5310	directIF	Negative
Pipistrellus pipistrellus	Wallonië	1367	directIF	Negative
Pipistrellus pipistrellus	Wallonië	1360	directIF	Negative
Pipistrellus pipistrellus	Wallonië	5032	directIF	Negative
Pipistrellus pipistrellus	Wallonië	1300	directIF	Negative
Pipistrellus pipistrellus	Wallonië	1450	directIF	Negative
Pipistrellus pipistrellus	Vlaanderen	1620	directIF	Negative
Pipistrellus pipistrellus	Wallonië	6220	directIF	Negative
Myotis mystacinus	Wallonië	1380	directIF	Negative
Eptesicus serotinus	Wallonië	6740	directIF	Negative
Eptesicus serotinus	Wallonië	6740	PCR	Positive
Eptesicus serotinus	Vlaanderen	8000	directIF	Negative
Eptesicus serotinus	Vlaanderen	8000	PCR	Negative
Pipistrellus pipistrellus	Brussel	1000	directIF	Negative
Pipistrellus pipistrellus	Brussel	1000	directIF	Negative
Pipistrellus pipistrellus	Brussel	1000	directIF	Negative
Pipistrellus pipistrellus	Brussel	1000	directIF	Negative
Pipistrellus pipistrellus	Brussel	1000	directIF	Negative
Pipistrellus pipistrellus	Brussel	1000	directIF	Negative
Pipistrellus pipistrellus	Brussel	1000	directIF	Negative
Pipistrellus pipistrellus	Brussel	1000	directIF	Negative
Pipistrellus pipistrellus	Brussel	1000	directIF	Negative
Pipistrellus pipistrellus	Brussel	1000	directIF	Negative
Pipistrellus pipistrellus	Brussel	1000	directIF	Negative
Pipistrellus pipistrellus	Brussel	1000	directIF	Negative
Pipistrellus pipistrellus	Wallonië	1300	PCR	Negative
Rousettus aegyptiacus?	Vlaanderen	2018	directIF	Negative
Pipistrellus pipistrellus	Wallonië	1348	PCR	Negative
Eptesicus serotinus	Wallonië	5540	directIF	Negative
Eptesicus serotinus	Wallonië	5540	PCR	Negative
Myotis nattereri	Vlaanderen	3640	directIF	Negative
Myotis nattereri	Vlaanderen	3640	PCR	Negative
Eptesicus serotinus	Wallonië	5024	PCR	Negative
Eptesicus serotinus	Wallonië	5024	directIF	Negative
Pipistrellus pipistrellus	Vlaanderen	1850	directIF	Negative
Pipistrellus pipistrellus	Wallonië	1410	PCR	Negative
Pipistrellus pipistrellus	Vlaanderen	3960	directIF	Negative
Pipistrellus pipistrellus	Vlaanderen	3790	directIF	Negative
Pipistrellus pipistrellus	Wallonië	5310	PCR	Negative
Pipistrellus pipistrellus	Wallonië	1300	PCR	Negative
Pipistrellus pipistrellus	Wallonië	1348	PCR	Negative
Pipistrellus pipistrellus	Brussel	1000	directIF	Negative
Pipistrellus pipistrellus	Wallonië	5500	PCR	Negative
Pipistrellus pipistrellus	Refused	Unknown		
Pipistrellus pipistrellus	Wallonië	4920	PCR	Negative
Plecotus sp.	Wallonië	6723	directIF	Negative
Pipistrellus pipistrellus	Wallonië	1495	PCR	Negative
Pipistrellus pipistrellus	Wallonië	1400	PCR	Negative
Pipistrellus pipistrellus	Wallonië	5032	PCR	Negative
Pipistrellus pipistrellus	Wallonië	5032	PCR	Negative
Pipistrellus pipistrellus	Wallonië	5030	PCR	Negative
Pipistrellus pipistrellus	Wallonië	1320	PCR	Negative
Pipistrellus pipistrellus	Wallonië	1300	PCR	Negative
Plecotus sp.	Vlaanderen	9660	PCR	Negative
Pipistrellus pipistrellus	Brussel	1030	PCR	Negative
Pipistrellus pipistrellus	Wallonië	6987	PCR	Negative
Pipistrellus pipistrellus	Wallonië	1331	PCR	Negative

Pipistrellus pipistrellus	Wallonië	1390	PCR	Negative
Pipistrellus pipistrellus	Vlaanderen	9820	directIF	Negative
Pipistrellus pipistrellus	Vlaanderen	9820	PCR	Negative
Plecotus sp.	Vlaanderen	9900	PCR	Negative
Myotis daubentonii	Vlaanderen	9890	PCR	Negative
Plecotus sp.	Vlaanderen	9630	PCR	Negative
Eptesicus serotinus	Vlaanderen	9000	PCR	Negative
Eptesicus serotinus	Unknown	Unknown	PCR	Negative
Pipistrellus pipistrellus	Brussel	1050	directIF	Negative
Pipistrellus pipistrellus	Brussel	1050	PCR	Negative
Pipistrellus pipistrellus	Vlaanderen	3511	directIF	Negative
